# Increased Neuronal Differentiation Efficiency in High Cell Density-Derived Induced Pluripotent Stem Cells

**DOI:** 10.1155/2019/2018784

**Published:** 2019-12-04

**Authors:** Sumitra Srimasorn, Matthias Kirsch, Susanne Hallmeyer-Ellgner, Dirk Lindemann, Alexander Storch, Andreas Hermann

**Affiliations:** ^1^Department of Neurology, Technische Universität Dresden, Dresden, Germany; ^2^Department of Neurosurgery, Technische Universität Dresden, Dresden, Germany; ^3^CRTD/DFG-Center for Regenerative Therapies Dresden, Technische Universität Dresden, Dresden, Germany; ^4^Institute of Virology, Medical Faculty “Carl Gustav Carus”, Technische Universität Dresden, Dresden, Germany; ^5^Department of Neurology, University Medical Center Rostock, University of Rostock, 18147 Rostock, Germany; ^6^German Center for Neurodegenerative Diseases (DZNE) Rostock/Greifswald, 18147 Rostock, Germany; ^7^Center for Transdisciplinary Neurosciences Rostock (CTNR), University Medical Center Rostock, University of Rostock, 18147 Rostock, Germany; ^8^Translational Neurodegeneration Section “Albrecht-Kossel”, Department of Neurology, University Medical Center Rostock, University of Rostock, 18147 Rostock, Germany

## Abstract

Human pluripotent stem cells (hPSCs), including induced pluripotent stem cells (iPSCs), provide access to hard-to-obtain cells for studies under physiological and disease conditions. For the study of neurodegenerative diseases, especially sporadic cases where the “disease condition” might be restricted towards the neuroectodermal lineage, obtaining the affected neurons is important to help unravel the underlying molecular mechanism leading to the diseases. Although differentiation of iPSCs to neural lineage allows acquisition of cell types of interest, the technology suffers from low efficiency leading to low yield of neurons. Here, we investigated the potential of adult neuroprogenitor cells (aNPCs) for iPSC derivation and possible confounders such as cell density of infected NPCs on their subsequent neuronal differentiation potential from reprogrammed cells under isogenic conditions. Characterized hiPSCs of defined cell densities generated from aNPCs were subjected to neuronal differentiation on PA6 stromal cells. The results showed that hiPSC clones obtained from low seeding density (iPSC-aNPC_Low_) differentiated less efficiently compared to those from higher density (iPSC-aNPC_High_). Our findings might help to further improve the yield and quality of neurons for *in vitro* modelling of neurodegenerative diseases.

## 1. Introduction

The study of cellular and molecular attributes of neurodegenerative diseases has been limited by the insufficiency to access diseased cells. Obtaining cells or tissues from affected individuals is not only highly invasive and often leads to death of the neurons but since these patient-specific cells are at the late stage of the disease, it further restricts the understanding of the onset mechanisms. Human embryonic stem cells (hESCs) have been shown to efficiently differentiate into functional neurons and glia in a manner similar to *in vivo* development [1–4]. These cells have been proposed as a tool for investigation of neurological diseases. Human induced pluripotent stem cells (hiPSCs), hESCs-like cells, have emerged as an alternative source, overcoming the drawbacks of hESCs which lack the disease conditions of the individual, thus allowing direct examination of diseased cells for pathological studies and drug screening (review by [5, 6]).

Human iPSCs were first generated from skin fibroblast by a set of core pluripotent transcription factors [7]. Since then, studies including the use of different somatic cells as starting cell source, transgene-free methods, and reduction or replacement of transcription factors have been performed to improve the quality of hiPSCs generated [8–13]. Despite its pluripotent nature, some of the fundamental questions that arose are (1) whether hiPSCs can differentiate efficiently into target cell lineage, like neural cells and (2) if these iPSCs-derived cells are functional. Hu et al. conducted the study where they compared the neural differentiation potential of hiPSCs with hESCs revealing that hiPSCs undergo the same time course and transcriptional network as hESCs during neural differentiation. Furthermore, they showed that hiPSCs can undergo neuro- and gliogenesis to generate functional neurons and glia *in vitro* [14]. This study further indicates the valuable nature of hiPSCs for regenerative medicine.

Although the supply of neurons derived from hiPSCs, including disease-specific neurons, is unlimited, the differentiation efficiency is lower and more variable when compared to hESCs-derived neuronal cells [14]. Loehle et al. showed that neuronal differentiation efficiency, as well as reprogramming efficiency, in murine cells decreases when the number of transcription factors was decreased [9]. On the contrary, we recently showed that reducing reprogramming factors in human cells does not alter the neuroectodermal differentiation efficiency [15]. Although the stepwise conversion of hiPSCs to neurons with increased homogeneity has been reported [10, 16], the differentiation efficiency was dependent on the “survival of the fittest” stem cells differentiated from iPSCs. This shows that techniques other than altering the number of transcription factors or culture conditions are important for improving neuronal differentiation efficiency from hiPSCs.

Here, we show that the cell density of infected adult neuroprogenitor cells (aNPC) plays a role in the efficacy of subsequent neuronal differentiation. To rule out germ layer effects, we compared isogenic hiPSC lines from aNPCs of the same donor seeded at low (iPSC-aNPC_Low_) and high (iPSC-aNPC_High_) cell densities. Our results show that neuronal differentiation efficiency is significantly higher in iPSCs obtained from high density in comparison to low density. This finding might help improve the yield of patient-specific neurons and facilitate high-throughput/high-content studies of underlying mechanisms and potential drug discoveries.

## 2. Materials and Methods

### 2.1. Preparation and Culture of Tissue Obtained from the Adult Human Brain

Cortical white matter tissues were obtained from routine epilepsy surgery procedures with informed consent from all donors. All procedures were in accordance to the Helsinki convention and with approval from the Ethical Committee of Technische Universität Dresden (EK No. 45022009, 47032006). ANPCs were derived as previously described [15, 17–20]. In brief, to homogenize, tissues were minced with single-use scalpels followed by incubation in 2.5 mg/ml Trypsin solution (Sigma-Aldrich) at 37°C for 15 min. Tissues were centrifuged at 230 g for 4 min and incubated at 37°C for 15 min in 0.04 mg/ml DNase I (Sigma-Aldrich) solution at equal volume to the sample. After centrifugation, homogenized tissues were resuspended in N5 medium (DMEM-High glucose and F12-Glutamax at 1 : 1, 2% N2 supplement (Life Technologies), 5% FCS (Biochrome), 100 U/ml penicillin and 100 *μ*g/ml streptomycin (1% P/S; Life Technologies), and 35 *μ*g/ml pituitary extract (Life Technologies)). Medium was supplemented with 1 : 1000 mLIF (Merck Chemicals GmbH) and 1 : 500 bFGF2: EGF mixture (at 1 : 1 ratio; Sigma-Aldrich). Tissues were titrated using 1 ml tip followed by fire-polished glass Pasteur pipette to dissociate and cultured as suspension at 3% O_2_, 37°C, and 5% CO_2_. Growth factors were added every other day without medium change. Three weeks after dissociation, suspension cultures were passed through 100 *μ*m cell strainer. The pass through was cultured separately from the cells collected in the strainer, both at 3% O_2_, 37°C, and 5% CO_2_.

### 2.2. Preparation of Feeder Cells

SIM thioguanine/ouabain-resistant MEF (or shortly STO) cell line was purchased from ATCC (Catalogue No. CRL-1503) and thawed and cultured following the instructions provided (refer to Supplementary Methods for detail). For inactivation, cells were either thawed or passaged onto 150 cm^2^ flask coated with 0.1% *w*/*v* gelatine solution. At confluency, medium was replaced to fresh medium containing 10 *μ*g/ml mitomycin c (MMC; Tocris Bioscience) and incubated for 2 h at 37°C, 5% CO_2_. Inactivated cells were trypsinized and centrifuged at 125 g for 4 min. The pellet was resuspended in cold growth medium and counted using Neubauer chamber. Inactivated STO cells (referred to as STO feeder hereon) were resuspended in DMEM-High glucose, 10% FBS (Sigma-Aldrich), 1% P/S, and 10% DMSO (Sigma-Aldrich) at final density of 3 × 10^6^ cells/ml and stored at -80°C before usage.

### 2.3. Virus Generation

Lentivirus was produced as described [21] in HEK293T cells by cotransfection of 3.19 *μ*g of lentiviral vector (obtained from Prof. Dr. Axel Schambach, Department of Experimental Hematology, Hannover Medical School, Germany) and helper plasmids (7.66 *μ*g pMDLg/pRRE, 3.19 *μ*g pRSV-Rev, and 0.96 *μ*g pMD2.G) in 10 cm^2^ culture dish using 45 *μ*g of polyethylenimine (Sigma-Aldrich). Medium was changed 4 to 6 h later to fresh N5 medium and incubated for further 24 h. Virus supernatant was harvested and filtered through 0.45 *μ*m PVDF filter (Millipore) and either used directly for transduction or aliquoted and stored at -80°C.

### 2.4. iPSC Generation and Maintenance

IPSC generation, propagation, and characterization was principally performed as reported recently [9, 15, 22–25]. To generate iPSCs, neurospheres were dissociated to single cells one day prior to transduction. For that, spheres were pelleted by gravity, washed with warm DPBS, and resuspended in 1 ml Accutase solution (Sigma-Aldrich) and incubated for 15 min at 37°C. From 10 min time point, spheres were titrated using fire-polished glass Pasteur pipette. Single cells were resuspended in N5 medium with growth factors and plated as suspension culture in 6 cm dish overnight at 3% O_2_, 37°C, and 5% CO_2_. For transduction, single-celled aNPCs were centrifuged at 250 g for 4 min and resuspended in 5 ml virus supernatant mixture (1 : 1 of virus supernatant and fresh N5 medium) supplemented with 5 ng/ml bFGF2 and 4 *μ*g/ml protamine sulphate (Sigma-Aldrich). Cells were incubated as suspension culture for 24 h at 37°C, 5% CO_2_, and 21% O_2_. The next day, pelleted cells were suspended in fresh N5 medium and plated at 1.75 × 10^4^ and 5 × 10^4^ cells per 10 cm dish on STO feeder supplemented with 5 ng/ml bFGF2 and 1 mM valproic acid (Sigma-Aldrich). Half medium change was performed the next day by replacing with iPSC medium (Knockout™ DMEM with 20% Knockout™ Serum Replacement, 1% nonessential amino acids (NEAA), 1% P/S (all Life Technologies), 0.1 mM *β*-mercaptoethanol, 5 mg/ml heparin (Sigma-Aldrich)) supplemented with 5 ng/ml bFGF2 and 1 mM valproic acid. From next day onwards, medium was completely replaced every day with iPSC medium supplemented with 5 ng/ml bFGF2 and 1 mM valproic acid. Valproic acid was withdrawn once colonies were observed (approximately 4-6 days post transduction). Methods for characterization of pluripotency and transgene silencing are provided in Supplementary Methods.

### 2.5. Neuronal Differentiation

IPSCs from passages between 10 and 25 were subjected to neuronal differentiation on PA6 stromal cells as described previously [9, 15, 26]. Briefly, PA6 stromal cells were plated on 4-well plates 24 h prior to differentiation. Medium was changed to Glasgow Minimum Essential Medium (GMEM) supplemented with 10% Knockout Serum Replacement, 2 mM L-glutamine, 1 mM sodium pyruvate, 1× NEAA (all Life Technologies), 0.1 mM *β*-mercaptoethanol, and 1% P/S with 10 *μ*M Y27632 (Tocris Bioscience) two hours before plating of the iPSCs. For plating of iPSCs, one well of 6-well plate was incubated with 1 mg/ml collagenase type IV (Life Technologies) for 5 min to lift the colonies. Three or four colonies for each clone were picked into 1.5 ml Eppendorf tube containing iPSC medium and centrifuged at 100 g for 1 min. Colonies were then resuspended with the pre-incubated medium from the 4-well plates and plated on one complete 4-well plate. Medium was changed on day 4 and every other day following that. From day 14 onwards, medium change was performed with DMEM supplemented with 1% N2, 2 mM L-glutamine, 1 mM sodium pyruvate, 0.1 mM NEAA (all Life Technologies), 0.1 mM 2-mercaptoethanol, and 1% P/S. Colonies were fixed and analysed via immunocytochemistry on day 28. Colonies were assessed as positive when at least one cell within the colony showed positive marker expression.

### 2.6. Immunocytochemical Analysis

Immunostaining was performed according to Hermann et al. with modifications [15]. Briefly, colonies in the wells were fixed with 4% paraformaldehyde solution for 12 min after washing with DPBS once. Cells or colonies were permeabilized using 0.02% Triton-X (Fisher Scientific) in DPBS for 10 min. Wells were washed 2-3 times with DPBS and incubated in blocking solution (1% *w*/*v* Fraction V bovine serum albumin and 5% *v*/*v* donkey serum in DPBS containing 0.3 M *w*/*v* glycine and 0.02% (*v*/*v*) Triton-X 100, pH 7.4) for 1 h at room temperature (RT). Primary antibodies diluted in blocking solution (Supplement [Supplementary-material supplementary-material-1]) were added and incubated at 4°C overnight. After washing with DPBS four times, colonies were incubated with secondary antibodies (Supplement [Supplementary-material supplementary-material-1]) in dark at RT for 1 h. Hoechst 33342 (7.5 *μ*g/ml in DPBS; Invitrogen) was used for nuclei staining and the coverslips were mounted on glass slides with Fluoromount-G® (Southern Biotec). Microscopy analyses were performed using an inverted fluorescence microscope (Observer.Z1; Zeiss).

### 2.7. Statistical Analysis

For multiple comparison of means between groups, one-way analysis of variance (ANOVA) with Bonferroni's multiple comparison test was conducted. Statistical significance was considered when *p* < 0.05. Statistical processing was performed using GraphPad Prism software. Experiments were repeated four times for each set of measurements (*n* = 4).

## 3. Results

### 3.1. Induced Pluripotent Stem Cells from Adult Human Neuroprogenitor Cells

**Figure 1:**
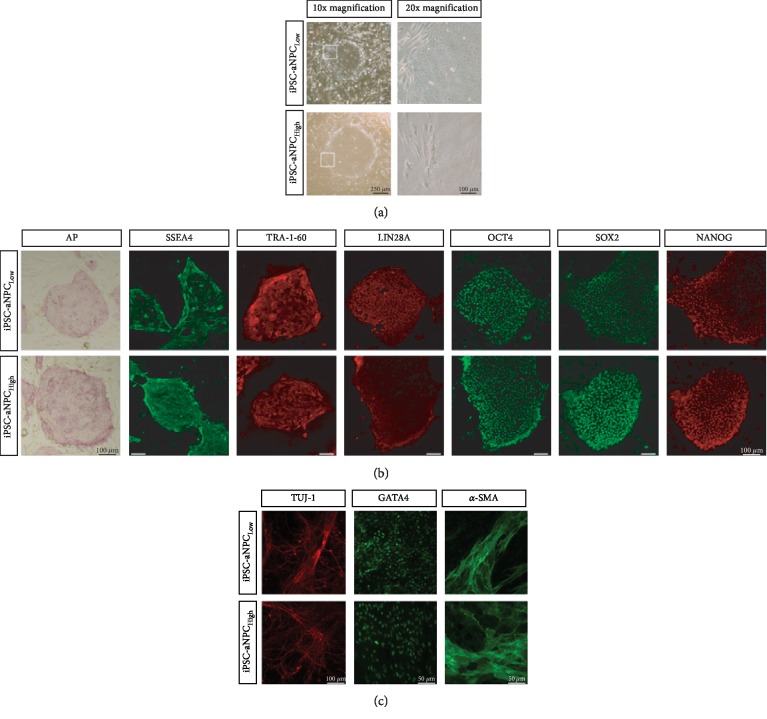
Characterization of induced pluripotent stem cells (iPSCs) generated from adult human neuroprogenitor cells (aNPCs). Both iPSC-aNPC_Low_ and iPSC-aNPC_High_ show (a) round colonies with no spontaneous differentiation observed around the border of the colonies. Scale bar is 250 *μ*m and 100 *μ*m for 10x and 20x magnifications, respectively. (b) Representative images of clones stained for surface markers including AP, SSEA4, and Tra 1-60 and cytoplasmic and transcription factors including LIN28A, OCT4, SOX2, and NANOG which are all markers for pluripotency. Clones are positive for all markers tested. Scale bar represents 100 *μ*m. (c) *In vitro* germ layer differentiation of iPSC clones from both low and high densities stained for TUJ1, GATA4, and *α*-SMA representing ecto-, endo-, and mesoderm markers, respectively. Representative images are shown here. Scare bars are 100 *μ*m and 50 *μ*m.

We have previously characterized sphere cultures obtained from white matter and hippocampus and showed that these cells are multipotent neuroprogenitor cells [17–20, 27]. To generate iPSCs, polycistronic lentiviral vector containing Yamanaka factors was used for production of virus supernatant [21]. Dissociated aNPC spheres were transduced for 24 h after which the cells were plated at density of either 1.75 × 10^4^ (low) or 5 × 10^4^ (high) cells per 10 cm dish. The reprogramming efficiencies were 0.017% and 0.014% for low and high density, respectively. Primary selection of iPSCs was based on the morphology followed by confirmation of silenced exogenous and activation of endogenous transcription factors (Figure 1(a) and [Supplementary-material supplementary-material-1], respectively). All clones showed low expression of residual exogenous *OCT4* expression. In parallel, endogenous *OCT4*, *NANOG*, and *LIN28A* were expressed at levels similar to human ESCs (H9) ([Supplementary-material supplementary-material-1]). After expansion, iPSCs from each cell density were characterized for pluripotency via immunostaining against surface markers including alkaline phosphatase (AP), SSEA4, and TRA-1-60 and cellular markers LIN28A, OCT4, SOX2, and NANOG (Figure 1(b)). *In vitro* germ layer differentiation showed positive expression of markers for ecto- (TUJ-1), endo- (GATA4), and mesoderm (ɖ-smooth muscle actin (SMA)) (Figure 1(c)). Confirmed iPSC clones obtained from low (iPSC-aNPC_Low_) and high densities (iPSC-aNPC_High_) were then subjected to neuronal differentiation on PA6 stromal cells (refer to Materials and Methods).

### 3.2. Neuronal Differentiation of Induced Pluripotent Stem Cells from High-Density Cultures Showed Higher Efficiency

**Figure 2:**
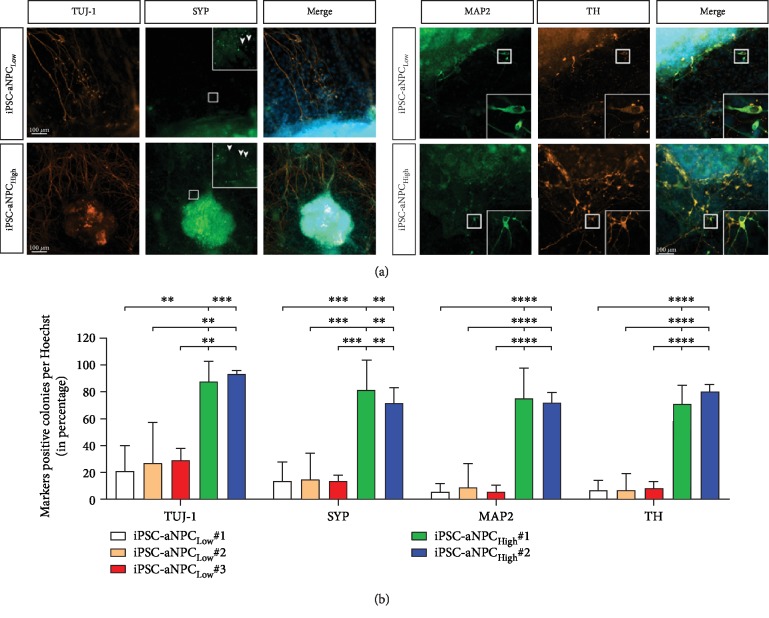
Neuronal differentiation of iPSC-aNPCs. Representative images of clones from iPSC-aNPC_Low_ and iPSC-aNPC_High_ subjected to differentiation on PA6 stromal cells stained for (a) TUJ1 and SYP (arrowheads) for immature neuron and presynaptic markers and for MAP2 and TH for mature neuron and dopaminergic neuron markers, respectively. Scale bar represents 100 *μ*m. Insets show higher magnifications. (b) Quantification of marker-positive colonies as a percentage of Hoechst-positive colonies for TUJ1 (*F* value: 15.56; *p* value < 0.0001), SYP (*F* value: 18.14; *p* value < 0.0001), MAP2 (*F* value: 29.13; *p* value < 0.0001), and TH (*F* value: 61.4; *p* value < 0.0001). Bar shows mean ± SEM for four independent experiments. ^∗∗^*p* < 0.01, ^∗∗∗^*p* < 0.001, and ^∗∗∗∗^*p* < 0.0001.

In order to check for possible differences in neurectodermal differentiation capacity, we decided to use the protocol for neuronal differentiation on PA6 stromal cells [9]. The advantage of this protocol is the direct differentiation from the iPSC without intermediate stages which might influence differentiation efficiency by the “survival of the fittest” stem cells differentiated from iPSCs [10, 16]. Three iPSC-aNPC_Low_ and two iPSC-aNPC_High_ isogenic clones (derived from the same patient and same passage aNPCs) were plated on PA6 stromal cells to induce midbrain dopaminergic neuron differentiation [9, 15, 26]. During the differentiation, iPSC clones formed islands of colonies, allowing for the identification of single colonies. Four weeks of post differentiation induction, colonies were fixed and immunostained for immature (TUJ-1) and mature (MAP2) neuronal markers (Figure 2(a)). Quantification of the markers showed significant difference in TUJ-1 expression between the groups (*F*(4, 15) = 15.56, *p* < 0.001, one-way ANOVA), but no significant differences within iPSC-aNPC_Low_ clones (20.78 ± 9.7, 26.28 ± 15.27, and 28.46 ± 4.73; clone #1, #2, and #3, respectively, *p* > 0.9999) and iPSC-aNPC_High_ clones (87.32 ± 7.7 and 93.07 ± 1.44; clone #1 and #2, respectively, *p* > 0.9999) (Figure 2(b) and Supplement [Supplementary-material supplementary-material-1]). However, when comparing between iPSC-aNPC_Low_ and iPSC-aNPC_High_, significant differences were observed. IPSC-aNPC_Low_ #1 showed significantly lower TUJ-1 expression when compared to both iPSC-aNPC_High_ #1 and #2 clones (20.78 ± 9.7 vs. 87.32 ± 7.7 and 93.07 ± 1.44; *p* = 0.0011 and 0.0005, respectively) (Figure 2(b)). Similarly, iPSC-aNPC_Low_ #2 and #3 were also significantly less efficient than iPSC-aNPC_High_ #1 and #2 (Figure 2(b); refer to Supplement [Supplementary-material supplementary-material-1] for detailed analysis). For MAP2 expression, iPSC-aNPC_High_ showed significant differences to iPSC-aNPC_Low_ with the former differentiating more efficiently (Figure 2(b); refer to Supplement [Supplementary-material supplementary-material-1] for detailed statistics).

Next, to show that these neurons become mature and develop functional prerequisites, colonies were stained for synaptophysin (SYP), a synaptic vesicle glycoprotein known to play role in synaptic transmission in neurons, also used as a marker for presynaptic terminal and synaptodendritic function [28, 29]. Neurons from all clones stained positive for SYP, as shown by the dot-like pattern along the neurites (Figure 2(a), white arrowheads). When quantified, significant difference was noticed between the groups (*F*(4, 15) = 18.14, *p* < 0.0001) (Figure 2(b)). All three iPSC-aNPC_Low_ clones expressed significantly lower SYP than iPSC-aNPC_High_ #1 (*p* = 0.0003 for all comparisons) and iPSC-aNPC_High_ #2 (*p* < 0.0016) clones (Figure 2(b); refer to Supplement [Supplementary-material supplementary-material-1] for detailed statistics).

The neuronal differentiation protocol used has been reported to yield midbrain dopaminergic neurons, which is crucial for Parkinson's disease studies, for example, [26]. Therefore, colonies were stained for tyrosine hydroxylase (TH) (Figure 2). Although clones from both low and high densities stained positive for TH, a statistical difference between groups existed as determined by one-way ANOVA (*F*(4, 15) = 61.4, *p* < 0.0001) (Figure 2(b)). Again, no significant differences were noticed within iPSC-aNPC_Low_ clones (6.59 ± 3.33, 6.41 ± 6.41, and 7.84 ± 2.6; clones 1, 2, and 3, respectively; *p* > 0.9999 for all comparisons) and iPSC-aNPC_High_ clones (70.63 ± 6.99 and 79.96 ± 2.68; clones 1 and 2, respectively; *p* > 0.9999 for all comparisons). Conversely, significantly high expression of TH+ colonies was observed in iPSC-aNPC_High_ clones compared to iPSC-aNPC_Low_ clones (*p* < 0.0001 for all comparisons) (Figure 2(b); refer to Supplement [Supplementary-material supplementary-material-1] for detailed statistical analysis). No positive expression of the markers tested was observed in non-reprogrammed aNPCs subjected to differentiation on PA6 stromal cells (Supplement [Supplementary-material supplementary-material-1]).

The differentiation experiments were carried out between passages 10 and 25 of iPSCs. We noticed no difference in the differentiation efficiency in iPSC-aNPC_High_ clones but did observe a not significant decrease in differentiation efficiency in iPSC-aNPC_Low_ clones at higher passages (data not shown).

## 4. Discussion

Access to diseased cells like neurons is of necessity for the study of neurological diseases [13, 30]. Sporadic neurodegenerative diseases, which account for the majority, have been hypothesized to occur due to somatic mutations suggesting that the “disease condition” might be restricted in the neuroectodermal lineage [31, 32]. However, as brain samples from patients are almost inaccessible, understanding of the underlying cellular and molecular processes of such diseases remains a difficult task. The launch of the method to generate iPSCs, which possess potency similar to ESCs, has been a breakthrough in the study of human diseases and in the field of regenerative medicine [7, 33]. IPSCs can be generated from various somatic cells regardless of age and gender as well as health status. The latter is appealing as iPSCs obtained from diseased individuals retain the genetic and/or epigenetic information leading to the diseases, even after differentiation to desired cell types, making such cells an indispensable source for study of disease mechanisms [23–25]. Despite the many advantages, the use of iPSCs in the study of neurological diseases suffers from the variability in differentiation into neurons [9, 14, 30]. Epigenetic memory retention, for example, has been shown to cause reprogrammed cells to differentiate preferentially towards the germ layer of the original cell type [34, 35]. Similar lineage bias has also been reported amongst different lines of hESCs [36–38]. Through transcriptome analysis, Sun and colleagues recently revealed that different hESC lines possess different gene expression profiles with distinct enrichment in developmental processes, such as ectodermal, mesodermal, and endodermal development, which was consistent with their respective lineage bias [39]. Furthermore, they also showed that different hESC lines obviously utilize distinct mechanisms to maintain pluripotent state already influencing subsequent differentiation capacity.

The heterogeneity and relatively low yield of neurons, especially into specific subtypes, further limit researchers to perform various assays required for understanding the molecular and cellular causes of the disease. Using neuroectodermal cells as starting point for iPSC derivation might be of interest, particularly in sporadic neurodegenerative diseases, in which the “disease condition” might be restricted towards the neuroectodermal lineage. However, as adult NPCs are hard to obtain in sufficient numbers, data on possible influences of cell density of infected NPCs on their subsequent neuronal differentiation potential are beneficial.

In order to improve the efficiency of neuronal differentiation from iPSCs, various strategies have been implemented. These include the use of somatic cells from the same germ layer and/or the culture conditions like medium and addition of growth factors to support cell survival and maturation [6, 10, 13, 16, 30]. Interestingly, when using factor-reduced approaches for the derivation of iPSCs, the tissue of origin showed to influence the final differentiation capacity in murine but not human iPSCs [9, 15]. Here, we report that the seeding cell density of infected NPCs from adult human brain, prior to the reversion to pluripotent state, influences the neuronal differentiation efficiency. The number of colonies positive for neuronal markers TUJ-1 and MAP2 were significantly higher in iPSCs obtained from high density compared to those from low density (Figure 2). Furthermore, the number of TH-positive dopaminergic neurons are significantly increased in iPSC-aNPC_High_ clones (Figure 2(b)). These differences were independent of passage number used to start differentiation (between passages 10 and 25). This is different with previous reports where late passage iPSCs have been shown to (1) possess increased pluripotency and (2) to differentiate more efficiently in neurons [40, 41]. Thus, such variabilities are even more pronounced in case of differentiation into germ layers different from the originating cell source and once more underpin the value of the use of aNPC as starting material for modelling neurodegenerative diseases.

We noticed that not all mature neurons were dopaminergic neurons (Figure 2(a)). Kawasaki et al. also reported the presence of other subtypes of neurons obtained including GABAergic, cholinergic, and serotonergic neurons [26]. Whether iPSC-aNPC_Low_ differentiate more efficiently to a different subtype of neurons remains to be elucidated. However, the overall number of neurons was clearly reduced in low-density clones. Furthermore, the duration of the differentiation might also be another factor affecting the differentiation efficiency of iPSC-aNPC_Low_ clones, which might require longer time to yield similar levels of neurons as iPSC-aNPC_High_.

The Yap/Hippo pathway is known to regulate coordination of signalling networks that govern cell proliferation and apoptosis as well as stem cell renewal and differentiation [42–44]. Lian et al. reported the control of pluripotency in mammalian ESCs by the Hippo pathway in which YAP is found predominantly in the nucleus to regulate transcription. On the other hand, phosphorylation and cytoplasmic retention of YAP via activation of Hippo lead to differentiation of ESCs [45]. YAP overexpression has also been shown to increase reprogramming efficiency of fibroblast. Although the Hippo signalling cascade itself is well understood, the upstream regulators are still being unravelled and likely include cellular junction proteins such as E-cadherin and zona occludens protein [46].

Hsiao et al. recently showed that when the density of hESCs and hiPSCs increases, YAP localization in the nucleus decreases and, subsequently, its transcriptional activities [47]. The study further indicated that high-density culture condition enhances differentiation to neuroepithelial progenitor in a YAP-dependent manner. In the current study, cell density was a variable prior to the conversion into iPSCs. It has been reported that density may be sensed by the cytoskeleton like F-actin via its stabilization or disruption [48]. Whether Hippo/Yap cascade, sensed via cell density, affects the pluripotency and the further neuronal differentiation remains to be elucidated. We report here that cell density of transduced cells seeded before reversion plays a role in the differentiation efficiency of iPSCs into the neuronal lineage. As neuroprogenitor cells from adult human are hard to obtain in sufficient amount, the data on the influence of cell density on subsequent neuronal differentiation potential is mandatory and valuable.

## Data Availability

The authors declare that all data is included in the manuscript and supplement files.

## Supplementary Materials

Supplementary Materials Table S1: list of antibodies. Table S2: statistical analysis of marker tested as percentage of Hoechst-positive colonies. Table S3: list of primers. Supplementary Figure S1: characterization of iPSCs. Supplementary Figure S2: negative controls of neuronal differentiation markers investigated.
