# Supplementary material for: Increased Neuronal Differentiation Efficiency in High Cell Density-Derived Induced Pluripotent Stem Cells
Source: Stem Cells Int. 2019 Dec 4;2019:2018784. doi: 10.1155/2019/2018784 (PMC6913159; doi:10.1155/2019/2018784)
Supplement: Supplementary Materials — Table S1: list of antibodies. Table S2: statistical analysis of marker tested as percentage of Hoechst-positive colonies. Table S3: list of primers. Supplementary Figure S1: characterization of iPSCs. Supplementary Figure S2: negative controls of neuronal differentiation markers investigated. [file 2018784.f1.pdf]

Supplementary

Materials

Table S1. List of antibodies

a. **Primary antibodies**

Name	Host	Dilution	Manufacturer
α-Smooth Muscle Actin (α-SMA)	mouse	1:100	Dako North America, Inc., Carpinteria, USA (M0851)
GATA4	goat	1:1000	R&D Systems, Minneapolis, USA (AF2606)
GFAP	chicken	1:1000	Abcam®, Cambridge, USA (ab4674)
LIN28A	mouse	1:2000	Cell Signaling Technologies, Inc., Massachusetts, USA (5930)
MAP2 ab	mouse	1:500	BD Bioscience, Bedford, USA (556320)
Nanog	rabbit	1:100	Cell Signaling Technologies, Inc., Massachusetts, USA (35580)
OCT4	rabbit	1:1000	Abcam®, Cambridge, USA (ab19857)
SOX2	mouse	1:500	R&D Systems, Minneapolis, USA (MAB2018)
SSEA4	mouse	1:500	Abcam®, Cambridge, USA (ab16287)
Synaptophysin	mouse	1:400	Sigma Aldrich, St. Louis, USA (S5786)
Tyrosine Hydroxylase (TH)	rabbit	1:500	Pel Freeze Biologicals, Arkansan, USA (P40101)
Tra-1-60	mouse	1:500	Abcam®, Cambridge, USA (ab16288)

TUJ-1	mouse	1:2000	Covance, Princeton, USA (PRB-435P)

b. Secondary antibodies

**Name**	**Host**	**Dilution**	**Manufacturer**
Anti-chicken IgY FITC	donkey	1:500	EMD Millipore, Billerica, USA
Anti-goat IgG 488	donkey	1:500	Life Technologies Corporation, Carlsbad, USA
Anti-mouse IgG 488	donkey	1:500	Life Technologies Corporation, Carlsbad, USA
Anti-mouse IgG 594	donkey	1:500	Life Technologies Corporation, Carlsbad, USA
Anti-mouse IgM Rhodamine Red	goat	1:200	Jackson ImmunoResearch Laboratories, Inc., Pennsylvania, USA
Anti-rabbit IgG 488	donkey	1:500	Life Technologies Corporation, Carlsbad, USA
Anti-rabbit IgG 594	donkey	1:500	Life Technologies Corporation, Carlsbad, USA

Table S2. Statistical analysis of marker tested as percentage of Hoechst positive colonies.

a. **TUJ1 quantification.** One-way analysis of variance with Bonferroni's multiple comparison test shows that there is a significance difference with F (4, 15) = 15.56, P <0.0001. Shown below are the adjusted *p*-values from post-hoc a multiple comparison following Bonferroni's correction.

Bonferroni's multiple comparisons test	*Significance*	*Adjusted P Value*
iPSC-aNPC$_{LOW}$#1 vs. iPSC-aNPC$_{LOW}$#2	ns	>0.9999
iPSC-aNPC$_{LOW}$#1 vs. iPSC-aNPC$_{LOW}$#3	ns	>0.9999
iPSC-aNPC$_{LOW}$#1 vs. iPSC-aNPC$_{HIGH}$#1	**	0.0011
iPSC-aNPC$_{LOW}$#1 vs. iPSC-aNPC$_{HIGH}$#2	***	0.0005
iPSC-aNPC$_{LOW}$#2 vs. iPSC-aNPC$_{LOW}$#3	ns	>0.9999
iPSC-aNPC$_{LOW}$#2 vs. iPSC-aNPC$_{HIGH}$#1	**	0.0025
iPSC-aNPC$_{LOW}$#2 vs. iPSC-aNPC$_{HIGH}$#2	**	0.0011
iPSC-aNPC$_{LOW}$#3 vs. iPSC-aNPC$_{HIGH}$#1	**	0.0036
iPSC-aNPC$_{LOW}$#3 vs. iPSC-aNPC$_{HIGH}$#2	**	0.0015
iPSC-aNPC$_{HIGH}$#1 vs. iPSC-aNPC$_{HIGH}$#2	ns	>0.9999

b. **MAP2 quantification.** One-way ANOVA shows that there is a significance difference with F (4, 15) = 29.13, P <0.0001. Multiple comparison bonferroni's test shown below with adjusted *p*-values.

Bonferroni's multiple comparisons test	*Significance*	*Adjusted P Value*
iPSC-aNPC$_{LOW}$#1 vs. iPSC-aNPC$_{LOW}$#2	ns	>0.9999
iPSC-aNPC$_{LOW}$#1 vs. iPSC-aNPC$_{LOW}$#3	ns	>0.9999
iPSC-aNPC$_{LOW}$#1 vs. iPSC-aNPC$_{HIGH}$#1	****	<0.0001
iPSC-aNPC$_{LOW}$#1 vs. iPSC-aNPC$_{HIGH}$#2	****	<0.0001
iPSC-aNPC$_{LOW}$#2 vs. iPSC-aNPC$_{LOW}$#3	ns	>0.9999
iPSC-aNPC$_{LOW}$#2 vs. iPSC-aNPC$_{HIGH}$#1	****	<0.0001

iPSC-aNPC$_{LOW}$*#2 vs. iPSC-aNPC*$_{HIGH}$*#2*	****	<0.0001
iPSC-aNPC$_{LOW}$*#3 vs. iPSC-aNPC*$_{HIGH}$*#1*	****	<0.0001
iPSC-aNPC$_{LOW}$*#3 vs. iPSC-aNPC*$_{HIGH}$*#2*	****	<0.0001
iPSC-aNPC$_{HIGH}$*#1 vs. iPSC-aNPC*$_{HIGH}$*#2*	ns	>0.9999

c. **SYP quantification.** One-way ANOVA shows that there is a significance difference with F (4, 15) = 18.14, P <0.0001. Shown below are the adjusted *p*-values from post-hoc a multiple comparison following Bonferroni’s correction.

Bonferroni's multiple comparisons test	*Significance*	*Adjusted P Value*
iPSC-aNPC$_{LOW}$*#1 vs. iPSC-aNPC*$_{LOW}$*#2*	ns	>0.9999
iPSC-aNPC$_{LOW}$*#1 vs. iPSC-aNPC*$_{LOW}$*#3*	ns	>0.9999
iPSC-aNPC$_{LOW}$*#1 vs. iPSC-aNPC*$_{HIGH}$*#1*	***	0.0003
iPSC-aNPC$_{LOW}$*#1 vs. iPSC-aNPC*$_{HIGH}$*#2*	**	0.0013
iPSC-aNPC$_{LOW}$*#2 vs. iPSC-aNPC*$_{LOW}$*#3*	ns	>0.9999
iPSC-aNPC$_{LOW}$*#2 vs. iPSC-aNPC*$_{HIGH}$*#1*	***	0.0003
iPSC-aNPC$_{LOW}$*#2 vs. iPSC-aNPC*$_{HIGH}$*#2*	**	0.0016
iPSC-aNPC$_{LOW}$*#3 vs. iPSC-aNPC*$_{HIGH}$*#1*	***	0.0003
iPSC-aNPC$_{LOW}$*#3 vs. iPSC-aNPC*$_{HIGH}$*#2*	**	0.0015
iPSC-aNPC$_{HIGH}$*#1 vs. iPSC-aNPC*$_{HIGH}$*#2*	ns	>0.9999

d. **TH quantification against Hoechst positive colonies.** One-way ANOVA shows that there is a significance difference with F (4, 15) = 61.4, P <0.0001. Shown below are the adjusted *p*-values from post-hoc a multiple comparison following Bonferroni’s correction.

Bonferroni's multiple comparisons test	*Significance*	*Adjusted P Value*
iPSC-aNPC$_{LOW}$*#1 vs. iPSC-aNPC*$_{LOW}$*#2*	ns	>0.9999

iPSC-aNPC$_{LOW}$#1 vs. iPSC-aNPC$_{LOW}$#3	ns	>0.9999
iPSC-aNPC$_{LOW}$#1 vs. iPSC-aNPC$_{HIGH}$#1	****	<0.0001
iPSC-aNPC$_{LOW}$#1 vs. iPSC-aNPC$_{HIGH}$#2	****	<0.0001
iPSC-aNPC$_{LOW}$#2 vs. iPSC-aNPC$_{LOW}$#3	ns	>0.9999
iPSC-aNPC$_{LOW}$#2 vs. iPSC-aNPC$_{HIGH}$#1	****	<0.0001
iPSC-aNPC$_{LOW}$#2 vs. iPSC-aNPC$_{HIGH}$#2	****	<0.0001
iPSC-aNPC$_{LOW}$#3 vs. iPSC-aNPC$_{HIGH}$#1	****	<0.0001
iPSC-aNPC$_{LOW}$#3 vs. iPSC-aNPC$_{HIGH}$#2	****	<0.0001
iPSC-aNPC$_{HIGH}$#1 vs. iPSC-aNPC$_{HIGH}$#2	ns	>0.9999

Table S3. List of primers

Gene	Sequence	Product length
GAPDH (Glyceraldehyde-3-phospate)	5’-CTGGTAAAGTGGATATTGTTGCCAT-3’<br>5’-TGGAATCATATTGGAACATGTAAACC-3’	80 bp
LIN28A *(Lin-28 Homolog A)*	5’- GCAGCTTCTTCTCCGAACCAACCC-3’<br>5’- GGCGCAGCCACCTGCAAAC-3’	139 bp
NANOG *(Homeobox Protein NANOG)*	5’-AGCAATGGTGTGACGCAGAAGGC-3’<br>5’-TGGAAGGTTCCCAGTCGGGTTCA-3’	99 bp
OCT4 endogenous	5’-CTTCGCAAGCCCTCATTTCACCA-3’	154 bp

(Octamer-binding Transcription Factor 4)	5’-GTCCGAGGATCAACCCAGCCC-3’	
OCT4_KLF4 lentiviral	5’-GCATAGCAACTCTTCCGGA-3’<br>5’-GTTCCTCCCGCCATCTGTT-3’	194 bp
SOX2_cMYC lentiviral	5’-ATGAGCCAGCACTACCAGAG-3’<br>5’-GGTGAAGCTAACGTTGAGGG-3’	165 bp

Methods

STO cells culture

For thawing STO cells, culture flask was pre-incubated with STO growth medium (DMEM High-glucose with 10% Fetal Calf Serum (Sigma-Aldrich) and 1% P/S) for at least 15 min. Cells were rapidly thawed in 37°C water bath and the vial content was directly transferred to the 75cm^2 flask and placed at 37°C for attachment and growth. Medium change was performed every other day. Once confluent, cells were subcultured for 10 times prior to use as feeder cell layer. For passaging, cells were washed with DPBS and incubated with 0.05% Trypsin-EDTA solution at 37°C until cells detached. Cells were then collected with STO growth medium and gently pipetted to avoid clump formation. Resuspended cells were equally distributed into the flasks pre-incubated with growth medium, at the ratio of 1:10.

Gene expression analysis

Silencing of exogenous transcription factors was analysed using quantitative real-time polymerase chain reaction (qRT-PCR). For 2-step qRT-PCR, 100-1000 ng of RNA isolated from iPSCs using RNeasy kit (Qiagen) was transcribed to cDNA using QuantiTect Reverse Transcription Kit (Qiagen) following manufacturer’s protocol. qRT-PCR was performed using

QuantiTech SYBR® Green PCR Kit (Qiagen) with amplification reaction of 25 µl each (12.5 µl Quantitech SYBR-Green PCR-Master Mix, 1 µl cDNA (or water in case of NTC), 4 µl Primer (Forward and Reverse mixed) each at 5 pmol/µl, 7.5 µl RNA-free PCR grade water) in the presence of no template control (NTC). All qRT-PCR was performed using LightCycler® MX300PTM (Stratagene) and the setup includes: 15 min 95°C followed by 40 cycles of 15 s 94°C, 30 s 57°C, 30 s 72°C and 1 cycle of 1 min 95°C, 30 s 55°C and 30 s 95°C. For the analysis of transgene silencing evaluated from residual expression of exogenous *OCT4* relative to endogenous *OCT4*, qRT-PCR was conducted once and the quantification of gene expression level was performed using ΔΔCt method, where Ct is the cycle threshold. Samples were normalized to *GAPDH* (glyceraldehyde-3-phosphate-dehydrogenase; refer Table S3 for list of primers). For activation of endogenous pluripotent genes, expression level of endogenous *OCT4*, *NANOG*, and *LIN28A* were compared to levels of human ESC (H9; obtained from Prof. Elly Tanaka; CRTD, Dresden; Table S3). ΔCt values for iPSCs was obtained from one experiment while the average value obtained from two independent experiments for human ESC was used for comparison.

In vitro germ layer differentiation

Germ layer differentiation was conducted via embryoid body (EB) formation. For that, iPSCs colonies were detached using collagenase type IV (2 mg/ml; Life Technologies). Wells were incubated for 1-2 hrs and floating aggregates were collected and cultured in 6 cm suspension culture petri dish in iPSC medium supplemented with 5 µM Y27632, 10 µM SB431542 and 1 µM dorsomorphin (all from Tocris) for ectodermal differentiation or in iPSC medium supplemented 5 µM Y27632 for endo-/mesodermal differentiation. Two days later, medium was changed to same conditions without Y27632. On day 4, EBs were collected by gravity and resuspended in ectodermal differentiation medium (50% DMEM/F12, 50% Neurobasal containing 1:200 N2 supplement, 1:100 B-27 supplement without vitamin A (all Life Technologies), 1% penicillin/streptomycin/glutamine, 0.1% β-Mercaptoethanol and 1:500 BSA Fraction V (Life Technologies)) and plated on Matrigel™ (BD Bioscience) coated 4-well plate. For endo- and mesodermal differentiation, EBs were resuspended in endo-/mesodermal differentiation medium (77.9% DMEM (high glucose, Life Technologies), 20% FCS (Sigma Aldrich), 1% non-essential amino acids, 1% penicillin/streptomycin/glutamine and 0.1% β-

Mercaptoethanol (Life Technologies)) and plated on gelatine coated 4-well plate. Medium change was performed every other day for 14 days prior to fixation and immunocytochemical analysis.

Supplementary figures

Figure S1.

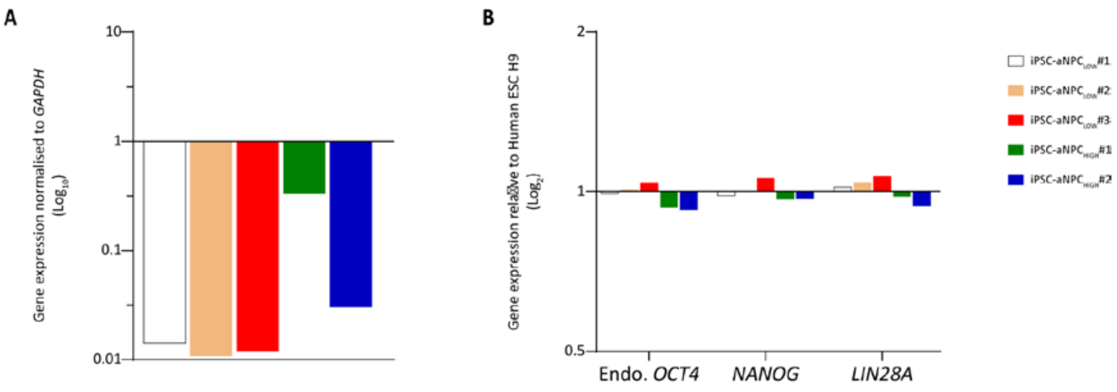

Supplementary Figure S1. Characterization of iPSCs. Gene expression analysis of iPSC-aNPC clones from low and high density showing low residual expression of exogenous *OCT4* (A). In parallel, the expression of endogenous pluripotent genes including *OCT4*, *NANOG*, and *LIN28A* were activated to similar level as that of human ESCs (H9; obtained from Prof. Elly Tanaka; CRTD, Dresden).

Figure S2.

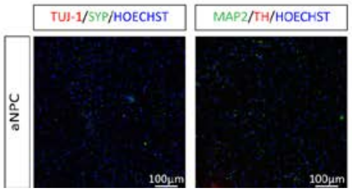

Supplementary Figure S2. Negative controls of neuronal differentiation markers investigated. aNPCs not subjected to reprogramming were plated on PA stromal cells and cultured in same conditions as iPSCs (Figure 2). aNPC here is age- and tissue type-match. Cells

do not express any of the markers tested (TUJ1, SYP, MAP2, and TH) showing that the protocol is specific for pluripotent stem cells. Scale bar represents 100 μm.
